# Assessment of Ebola virus disease preparedness in the WHO South-East Asia Region

**DOI:** 10.2471/BLT.16.174441

**Published:** 2016-09-22

**Authors:** Sirenda Vong, Reuben Samuel, Philip Gould, Hammam El Sakka, Bardan J Rana, Vason Pinyowiwat, Supriya Bezbaruah, Roderico Ofrin

**Affiliations:** a Health Security and Emergency Response Department, World Health Organization Regional Office for South-East Asia, Indraprastha Estate, Mahatma Gandhi Marg, New Delhi 110 002, India.

## Abstract

**Objective:**

To conduct assessments of Ebola virus disease preparedness in countries of the World Health Organization (WHO) South-East Asia Region.

**Methods:**

Nine of 11 countries in the region agreed to be assessed. During February to November 2015 a joint team from WHO and ministries of health conducted 4–5 day missions to Bangladesh, Bhutan, Indonesia, Maldives, Myanmar, Nepal, Sri Lanka, Thailand and Timor-Leste. We collected information through guided discussions with senior technical leaders and visits to hospitals, laboratories and airports. We assessed each country’s Ebola virus disease preparedness on 41 tasks under nine key components adapted from the WHO Ebola preparedness checklist of January 2015.

**Findings:**

Political commitment to Ebola preparedness was high in all countries. Planning was most advanced for components that had been previously planned or tested for influenza pandemics: multilevel and multisectoral coordination; multidisciplinary rapid response teams; public communication and social mobilization; drills in international airports; and training on personal protective equipment. Major vulnerabilities included inadequate risk assessment and risk communication; gaps in data management and analysis for event surveillance; and limited capacity in molecular diagnostic techniques. Many countries had limited planning for a surge of Ebola cases. Other tasks needing improvement included: advice to inbound travellers; adequate isolation rooms; appropriate infection control practices; triage systems in hospitals; laboratory diagnostic capacity; contact tracing; and danger pay to staff to ensure continuity of care.

**Conclusion:**

Joint assessment and feedback about the functionality of Ebola virus preparedness systems help countries strengthen their core capacities to meet the International Health Regulations.

## Résumé

**Objectif:**

Réaliser des évaluations de la préparation au virus Ebola dans des pays de la région Asie du Sud-Est de l'Organisation mondiale de la Santé (OMS).

**Méthodes:**

Sur les 11 pays de la région, neuf ont accepté d'être évalués. De février à novembre 2015, une équipe composée de membres de l'OMS et des ministères de la Santé ont effectué des missions de 4 à 5 jours au Bangladesh, au Bhoutan, en Indonésie, aux Maldives, au Myanmar, au Népal, au Sri Lanka, en Thaïlande et au Timor-Leste. Nous avons recueilli des informations lors de discussions guidées avec des directeurs techniques et lors de visites dans des hôpitaux, des laboratoires et des aéroports. Nous avons évalué la préparation de chaque pays au virus Ebola d'après 41 tâches relevant de neuf composantes clés, inspirées de la liste de contrôle de l'OMS pour faire face au virus Ebola de janvier 2015.

**Résultats:**

L'engagement politique en matière de préparation au virus Ebola était élevé dans tous les pays. La planification était plus avancée vis-à-vis des composantes qui avaient déjà été planifiées ou testées pour les pandémies de grippe: coordination multisectorielle et multi-niveaux, équipes d'intervention rapide multidisciplinaires, communication publique et mobilisation sociale, exercices dans les aéroports internationaux et formation au port d'équipements de protection individuelle. Les principales vulnérabilités étaient une mauvaise évaluation et communication des risques, des failles dans la gestion et l'analyse des données en vue de la surveillance, et des capacités limitées au niveau des techniques de diagnostic moléculaire. Dans de nombreux pays, la planification était limitée en cas de flambée du virus Ebola. D'autres tâches nécessitaient des améliorations, par exemple: les conseils aux voyageurs entrant dans le pays, la mise à disposition de chambres d'isolement adéquates, l'adoption de pratiques correctes de lutte contre la maladie, des systèmes de triage dans les hôpitaux, des capacités de diagnostic dans les laboratoires, la recherche des contacts et le paiement d'une prime de risque au personnel afin d'assurer la continuité des soins.

**Conclusion:**

L'évaluation conjointe et la communication au sujet de la fonctionnalité des systèmes de préparation au virus Ebola permettent aux pays de renforcer leurs capacités afin de respecter le Règlement sanitaire international.

## Resumen

**Objetivo:**

Llevar a cabo evaluaciones de la preparación ante el virus del Ébola en países de la región del sudeste asiático de la Organización Mundial de la Salud (OMS).

**Métodos:**

Nueve de once países de la región aceptaron ser evaluados. De febrero a noviembre de 2015, un equipo conjunto de la OMS y los ministerios de sanidad llevaron a cabo misiones de entre 4 y 5 días en Bangladesh, Bután, Indonesia, Maldivas, Myanmar, Nepal, Sri Lanka, Tailandia y Timor-Leste. Se recopiló información a través de conversaciones dirigidas con jefes técnicos y visitas a hospitales, laboratorios y aeropuertos. Se evaluó la preparación ante el virus del Ébola de cada país en 41 tareas con 9 componentes clave adaptados de la lista de preparación ante el ébola de la OMS de enero de 2015.

**Resultados:**

El compromiso político para la preparación ante el ébola era elevado en todos los países. La planificación era más avanzada para los componentes que habían sido previamente planificados o probados para pandemias de gripe: coordinación de varios niveles y sectores, equipos de respuesta rápida multidisciplinares, comunicación pública y movilización social, simulacros en aeropuertos internacionales y formación sobre equipos de protección individual. Entre las principales vulnerabilidades se encontraba una evaluación de riesgos y comunicación de riesgos poco adecuadas, lagunas en la gestión y el análisis de datos para el control de acontecimientos y una capacidad limitada en técnicas de diagnóstico molecular. Muchos países tenían una planificación limitada en caso de que resurgieran casos de ébola. Entre otras tareas que necesitaban mejorar se encontraban: asesoría a viajeros entrantes, salas de aislamiento adecuadas, prácticas de control de infecciones adecuadas, sistemas de clasificación en hospitales, capacidad de diagnóstico en laboratorios, localización de contactos y prima de peligrosidad para el personal para garantizar la continuidad de la atención.

**Conclusión:**

La evaluación conjunta y los comentarios sobre la funcionalidad de los sistemas de preparación ante el virus del Ébola ayudan a los países a fortalecer sus capacidades principales para cumplir el Reglamento Sanitario Internacional.

## ملخص

**الغرض:**

إجراء عدد من التقييمات للوقوف على مدى التأهب لمواجهة مرض فيروس الإيبولا في البلدان الواقعة ضمن منطقة جنوب شرق آسيا ضمن اختصاص منظمة الصحة العالمية.

**الطريقة:**

وافقت 11 بلدًا من البلدان الواقعة في المنطقة على الخضوع للتقييم. وقام فريق مشترك من منظمة الصحة العالمية ووزارات الصحة في كل بلد بالذهاب في مهمات تستغرق من 4 إلى 5 أيام إلى كل من بنغلاديش وبوتان وإندونيسيا وملديف وميانمار ونيبال وسري لانكا وتايلند وتيمور- لشتي، وذلك في الفترة من شهر شباط/فبراير وحتى شهر تشرين الثاني/نوفمبر من عام 2015. قمنا بجمع معلومات من خلال المناقشات التوجيهية مع بعضٍ من كبار المسؤولين المتخصصين ومن خلال الزيارات إلى المستشفيات والمختبرات والمطارات. قمنا بتقييم مدى التأهب لمواجهة مرض فيروس الإيبولا الذي تبديه كل بلد بإجراء 41 مهمة تتضمن تسعة عناصر أساسية معتمدة من قائمة الفحص الخاصة بمنظمة الصحة العالمية لقياس مدى التأهب لمواجهة الإيبولا في شهر كانون الثاني/يناير من عام 2015.

**النتائج:**

كان الالتزام بزيادة حالة التأهب لمواجهة الإيبولا مرتفعًا لدى الجهات السياسية في جميع البلدان. وكان التخطيط متقدمًا للغاية بالنسبة للعناصر التالية التي تم تخطيطها أو اختبارها مسبقًا لمواجهة وباء الإنفلونزا: التنسيق متعدد المستويات ومتعدد القطاعات؛ وفرق الاستجابة السريعة متعددة التخصصات؛ والاتصالات العامة والتعبئة الاجتماعية؛ وإجراء التدريبات في المطارات الدولية؛ والتدريب على استخدام معدات الوقاية الشخصية. تضمنت نقاط الضعف الرئيسية عدم كفاية أعداد تقييم المخاطر والإبلاغ عن المخاطر؛ ووجود فجوات في مجال إدارة البيانات وتحليلها لمراقبة الحدث؛ وتوفير تقنيات التشخيص الجزيئي بسعة محدودة. وقامت العديد من البلدان بعمليات تخطيط محدودة لمجموعة من الحالات المصابة بالإيبولا. وشملت المهام الأخرى التي تحتاج إلى التحسين ما يلي: تقديم النصيحة للمسافرين القادمين؛ وتوفير عدد كافٍ من غرف العزل؛ وإجراء الممارسات المناسبة لمكافحة العدوى؛ ووضع أنظمة فرز المصابين في المستشفيات؛ وزيادة السعة التشخيصية في المختبرات؛ وتتبع حالات الاختلاط؛ ومنح الموظفين بدل الخطر لضمان استمرارية أعمال الرعاية.

**الاستنتاج:**

إن التقييم وإبداء الملاحظات المشترك حول مدى فاعلية أنظمة التأهب لمواجهة فيروس الإيبولا يساعد البلدان في تعزيز قدراتها الأساسية على الالتزام باللوائح التنظيمية الصحية الدولية.

## 摘要

**目的:**

旨在评估东南亚地区世界卫生组织 (WHO) 成员国的埃博拉病毒病预防情况。

**方法:**

该地区 11 个国家中 9 个国家同意接受评估。2015 年 2 月至 11 月期间，WHO 及各国卫生部门组成了一个联合团队，在不丹、东帝汶、印度尼西亚、马尔代夫、孟加拉国、缅甸、尼泊尔、斯里兰卡、泰国开展了一项为期 4 到 5 天的任务。我们通过与高级技术领导人讨论、走访医院、实验室和机场收集信息，并针对 41 项任务对每个国家的埃博拉病毒预防情况进行了评估，其中 9 个关键组成部分来自于 WHO 2015 年 1 月的埃博拉预防清单。

**结果:**

所有国家政府都高度承诺对埃博拉进行预防。对于之前针对大流行性流感进行过计划或测试的组成部分实施最高级的计划：多层次和多部分协调；多学科快速反应小组；大众传播和社会动员；国际机场演习以及有关个人防护装备的培训。主要的不足包括风险评估和风险交流不足；针对事件监控的数据管理和分析不一致；以及分子诊断技术能力有限。许多国家应对埃博拉病例剧增的计划不足。需要提升的其他任务包括：向入境旅客提供建议；充足的隔离房间；恰当的感染控制措施；医院分诊系统；实验室诊断能力；接触者追踪；以及给予员工危险工作补贴以确保护理的连续性。

**结论:**

对埃博拉病毒预防体系的功能性进行联合评估和反馈，这有助于各国增强其符合国际卫生条例的核心能力。

## Резюме

**Цель:**

Провести оценку готовности к борьбе с вирусной лихорадкой Эбола в странах-участницах Всемирной организации здравоохранения (ВОЗ), расположенных в Юго-Восточной Азии.

**Методы:**

Согласие на проведение оценки выразили девять из 11 стран региона. С февраля по ноябрь 2015 года объединенная группа специалистов ВОЗ и представителей министерств здравоохранения осуществляла 4–5-дневные поездки в Бангладеш, Бутан, Индонезию, на Мальдивские острова, в Мьянму, Непал, Таиланд, Тимор-Лешти и Шри-Ланку. Информация была нами получена в ходе управляемых дискуссий с представителями высшего технического руководства этих стран и при посещении лабораторий, аэропортов и больниц. Готовность каждой из стран к борьбе с вирусной лихорадкой Эбола оценивалась исходя из 41 задачи в рамках 9 основных компонентов, которые были получены из списка контрольных вопросов по готовности к лихорадке Эбола, разработанных ВОЗ в январе 2015 года.

**Результаты:**

Идеологическая установка и подготовка к борьбе с лихорадкой Эбола оказалась высокой во всех странах. Что касается планирования, оно было наиболее развито в тех направлениях, которые уже ранее были спланированы или испытаны для случаев эпидемии гриппа: координация различных секторов и уровней, группы быстрого реагирования, имеющие многоплановый характер, связь с общественностью и социальная мобилизация, учения в международных аэропортах и обучение использованию средств индивидуальной защиты. Наиболее уязвимыми моментами оказались недостаточная оценка риска и недостаточная информированность о риске, были также выявлены недостатки в управлении данными и анализе при осуществлении контроля над событием, а также было выявлено, что применение молекулярных технологий диагностики недостаточно развито. Во многих странах планирование действий в случае вспышки лихорадки Эбола было ограниченным. К числу других задач, требующих улучшения, относятся следующие: рекомендации для лиц, перемещающихся внутри страны, достаточно оснащенные изоляторы, соответствующие практики ограничения распространения инфекции, системы сортировки пациентов в больницах, наращивание возможностей лабораторной диагностики, отслеживание контактов и дополнительная оплата работникам за риск для обеспечения непрерывного ухода за больными.

**Вывод:**

Совместная оценка и отзывы относительно функционирования систем готовности к вирусной лихорадке Эбола помогают странам укрепить ключевые возможности, необходимые для соблюдения международных медико-санитарных правил.

## Introduction

The 2013–2016 Ebola virus disease epidemic in West Africa was the largest ever reported, with 28 616 cases and 11 310 deaths as of June 2016.^1^ In August 2014, the World Health Organization (WHO) declared the epidemic a public health emergency of international concern, in accordance with the 2005 International Health Regulations (IHR).^2^ In January 2015, nine of the 11 countries from the WHO South-East Asia Region agreed to a joint assessment by WHO and ministries of health of their preparedness and operational readiness for Ebola virus disease.

The framework for the assessment were the key components and tasks proposed as indicators in the WHO consolidated Ebola preparedness checklist issued in January 2015.^3^ As the likelihood of Ebola virus disease introduction in the region was considered low, we focused mainly on minimum preparedness requirements and adapted the tasks according to the regional context. This report summarizes the findings of the country reviews in Bangladesh, Bhutan, Indonesia, Maldives, Myanmar, Nepal, Sri Lanka, Thailand and Timor-Leste.

## Methods

During February to November 2015 a series of 4–5 day missions were undertaken to each country by a joint assessment team comprising staff from WHO and the respective ministry of health (and in Thailand the United States Centers for Disease Control and Prevention as invited partners). Information was collected through guided discussions (1 or 2 days) with key ministry of health technical leaders (i.e. those responsible for ministry departments or divisions, and unit, branch or team leaders). The guided discussion technique^4^ aimed to elicit dialogue and exchanges between the assessors and participants, to review procedures and interdepartmental interactions and to analyse the functionality of the health emergency systems. Discussions continued with technical leaders during visits to specific settings: the country’s major international airport and its Ebola virus disease reference hospital and reference laboratory (1 day). We dedicated a half day to train the joint assessment team members and another half day to present preliminary findings to the same audience for clarification and to reach consensus (1 day). The final results and recommendations were summarized in the form of a report and presented to the health authorities of the country on the last day of the visit (1 day). WHO committed to monitoring the implementation of the recommendations. 

We designed a checklist to review each country’s preparedness activities and procedures on nine key components: A. Emergency planning; B. Risk assessment; C. Leadership and coordination; D. Surveillance and early warning; E. Laboratory diagnosis; F. Rapid investigation and containment; G. Infection control and clinical management; H. Communication; and I. Points of entry. Each assessment component comprised several tasks or activities (total 41) (Table 1). Each task addressed one of the three aspects of preparedness: what activities are currently operational for handling the threat of Ebola virus disease (currently functional activities); how prepared the country is for the introduction of an Ebola virus disease case (operational readiness); and how prepared the country is to face a wider outbreak of Ebola virus (surge capacity; Table 1).

**Table 1:** Scoring system used for assessing level of Ebola virus preparedness in the joint review of countries of the World Health Organization South-East Asia Region, February to November 2015

Task, by key component	Aspect of readiness assessed^a^	Level of functionality^b^			
		Not addressed (score 0)	Planned but not implemented (score 1)	Low functionality (score 2)	Complete response (score 3)
**A. Emergency planning for risk management**					
A1. Ebola virus disease preparedness plan implemented	Surge capacity	No plan	Ebola virus disease preparedness planned	Plan written, but incomplete implementation	Costed, risk-based approach, in line with WHO’s pandemic influenza preparedness plan
A2. Guidelines disseminated	Surge capacity	Not available	All Ebola virus disease-related WHO guidelines checked and read	WHO guidelines adapted and disseminated, but incompletely	WHO guidelines adapted and disseminated
A3. Funds release mechanism established	Operational readiness	No plan	Planned	Detailed procedures in place	Tested by past experience or simulation
A4. Staff bonus pay system in place for high-risk assignments	Operational readiness	No plan	Planned	Detailed procedures in place	Tested by past experience or simulation
A5. Contingency planning encouraged when appropriate	Surge capacity	No plan	Planned, with lists of agencies needing plan	Some contingency plans prepared	Tested by past experience or simulation
**B. Risk assessment processes**					
B1. Country-specific risk assessments conducted and capacity operational	Currently functional activities	No risk assessment conducted	At least one risk assessment reported produced	Risk assessments conducted, with regular updates, and disseminated	Risk assessment, with risk-based scenarios and recommendations documented
**C. Leadership and coordination in place and with surge capacity (multilevel and multisectoral)**					
C1. Ebola task force or Ebola committee established and operational	Operational readiness	Not mentioned	Strategies briefly mentioned	Multilevel and multisectoral approach established	Detailed strategies identified
C2. Membership of Ebola task force clearly described in plan, and updated	Operational readiness	Not mentioned	Mentioned	Terms of reference clear and reviewed	Tested by past experience or simulation
C3. Incidence management structure in place and operational	Operational readiness	Not in place	Mentioned in plan	Terms of reference clear and reviewed	Tested by past experience or simulation
C4. Emergency operation centre in place and operational	Operational readiness	Not in place	Roles and responsibilities to be defined	Detailed roles and responsibilities and communication and procedures in place	Tested by past experience or simulation
**D. Surveillance alert warning system**					
D1. Early warning system in place for haemorrhagic fever or Ebola virus disease cases	Currently functional activities	Not in place	Planned	Only indicator- or event-based surveillance enhanced	Indicator- or event-based surveillance enhanced for Ebola virus disease
D2. Indicator-based surveillance enhanced	Currently functional activities	Not enhanced	Planned	Instructions sent to hospitals and all health-care facilities	Staff trained extensively
D3. Event-based surveillance enhanced	Currently functional activities	No	Planned	Instructions sent to hospitals and all health-care facilities	Staff trained extensively
D4. Early warning reporting is timely and sensitive	Currently functional activities	Unknown	Limited surveillance infrastructure for timely reporting	Electronic data management system, but not timely and sensitive	Efficient, immediate reporting and analysis
D5. Rumours’ surveillance ready to operate	Currently functional activities	Not planned yet	Planned	Responsible department identified	Staff trained and tested
**E. Laboratory diagnosis**					
E1. Reference laboratories identified	Currently functional activities	No	At least one national reference laboratory identified	Staff trained for diagnostics	Quality assurance conducted
E2. Stand-by arrangements in place to ship samples from suspected Ebola cases for confirmatory testing	Operational readiness	No	With WHO collaborating centre and relevant airlines in place	Mechanism tested for other emergency infectious diseases in the past	Mechanism tested for Ebola virus disease as a drill
E3. Instructions on procedures for handling of infectious substances	Currently functional activities	Not distributed	Protocols online and readily available. Websites shared with all hospitals	Reference hospital laboratory staff trained	Hospital laboratory staff extensively trained
E4 Surge of public-health and clinical laboratories to meet planned needs	Surge capacity	No	Planned, but with no details	Planned, with some details	Detailed plan
**F. Rapid investigations, efficient contact tracing and containment**					
F1. Several rapid response teams on investigation identified	Operational readiness	No	Call-down list of rapid response team leaders available	Multisector team members identified and some trained	Rapid response team trained with drills
F2. Several rapid response teams on sampling procedures and on transport identified	Operational readiness	No	Call-down list of rapid response team leaders available	Multisector team members identified and some trained	Rapid response team trained with drills
F3. Several rapid response teams on personal protective equipment identified	Operational readiness	No	Call-down list of rapid response team leaders available	Multisector team members identified and some trained	Rapid response team trained with drills
F4. Several rapid response teams on Ebola case management identified	Operational readiness	No	Call-down list of rapid response team leaders available	Multisector team members identified and some trained	Rapid response team trained with drills
F5. Rapid response team ready for contact tracing	Operational readiness	No	Adapted strategy for contact tracing planned	Described	Rapid response team trained with drills
**G. Infection control and clinical management**					
G1. General awareness enhanced about hygiene and how to implement infection control in hospitals	Surge capacity	Planned	Instructions sent out	Training conducted	Tested or training with drills
G2. Isolation units and triage system for suspected Ebola cases in hospitals	Operational readiness	No	Planned, with call-down lists	Identified and equipped, with triage system	Training provided to all staff on infection control and prevention measures and waste management
G3. Surge increase in isolation rooms planned	Surge capacity	No	Planned	Procedures in place	Procedures tested
G4. Adequate capacity for clinical management of Ebola cases with haemorrhagic fever	Operational readiness	No	Planned	Planned but no procedures described	Detailed strategies and procedures
**H. Communication (dissemination mechanism, public information, social mobilization and risk communication)**					
H1. Communication coordination mechanism functional, involving all government sectors and other stakeholders	Operational readiness	No	Planned	In place, with partners and stakeholders identified	Tested
H2. Risk communication plan in place and operational in ministry of health	Operational readiness	No	Plan or strategy developed (centralized, different audience, partnership)	Experienced team or unit in place, with clear roles and responsibilities for Ebola risk communication materials	Training provided with simulation or drills conducted for Ebola virus disease
H3. Communication with the public and feedback mechanisms established	Surge capacity	No	Critical information materials planned (messages on Ebola virus disease available or functional procedures for review and validation)	Critical communication for use of information materials planned, with plan to engage community leaders	Mechanism in place to communicate with community leaders, and information materials readily available
H4. Procedures for information dissemination to all levels planned	Operational readiness	No	Mentioned but not implemented	Online websites developed but incomplete	Online websites developed and complete
H5. Advice to travellers to affected areas provided	Currently functional activities	No	Planned	Available from travel services	Available online and elsewhere (private and government agencies)
H6. Communication under International Health Regulations (IHR)	Operational readiness	No	IHR past experience	Trained	Exercises conducted
H7. Social mobilization planned	Surge capacity	No	Planned	Experience in engaging community leaders	Detailed plan made and experienced staff in place
**I. Points of entry**					
I1. Health emergency plan at airports ensured	Currently functional activities	No, but planned	In place	Training conducted extensively	Drills and simulations conducted or already tested, with updating
I2. Airport’s isolation room adequately equipped	Currently functional activities	No	Partially equipped	Fully equipped	Fully equipped in at-risk points of entry
I3. Airport's health teams on 24-hour 7-day stand-by, to assist travellers and ensure correct isolation	Currently functional activities	No	Procedures in place	Training provided	Procedures reviewed or tested
I4. Stand-by agreement with referral hospitals in place	Currently functional activities	No	Planned	Procedures described	Procedures reviewed or tested
I5. Communication procedures in place between health and airport authorities	Currently functional activities	No	Planned, with detailed mechanism described	In place	Tested
I6. Follow-up in place for at-risk travellers from Ebola-affected countries	Currently functional activities	No	Planned, with detailed mechanism described	In place	Monitoring system tested with at-risk travellers

WHO: World Health Organization.

^a^ Aspects of preparedness assessed by tasks were as follows: currently functional activities: activities currently operational in the country for Ebola virus disease detection; operational readiness: whether the country was ready for introduction of an Ebola virus disease case; surge capacity: how prepared the country was to face a wider Ebola virus outbreak.

^b^ Functionality of tasks was scored as follows: no structure in place or activities not addressed (score 0); activities planned but not implemented (score 1); activities in place but low evidence of functionality (score 2); or complete response, i.e. evidence of fully functional activities and readiness or planning for surge capacity (score 3).

Core questions were prepared to trigger guided discussion and contextual questions about the tasks (available from the corresponding author). To standardize analysis across countries and facilitate discussion between the joint assessment team members we scored the functionality of each task from 0 to 3: no structure in place or activities not addressed (score 0); activities planned but not implemented (score 1); activities in place but with low evidence of functionality (score 2); or complete response, i.e. evidence of fully functional activities and readiness or planning for surge capacity (score 3; Table 1). Readiness of a structure that was already in place was assessed on the level of training as follows: low evidence of functionality, when simple training such as a lecture or demonstration was carried out (score 2); or high evidence of functionality, if simulations were conducted regularly and reported to be followed by improvements (score 3). High surge capacity was defined as evidence of surge planning in terms of sufficient enrolment of trained staff and adequate space and supplies.^5^ Our assessments were based on documented evidence or participants’ descriptions of procedures.

The WHO Ethical Research Committee reviewed the programme methods and concluded that the activity did not qualify as research with human subjects.

## Results

### Planning

**Fig. 1:**
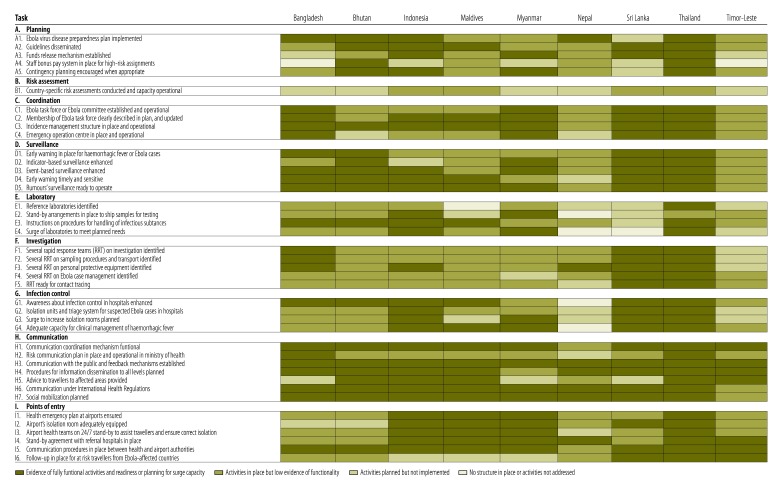
Status of tasks within key components in the review of Ebola virus disease preparedness in nine countries of the World Health Organization South-East Asia Region, February to November 2015

All nine countries had some level of preparedness for Ebola virus disease (Fig. 1). However, only seven of the countries had developed a specific, written Ebola virus disease preparedness plan (task A1), including four that had detailed a risk-based approach and some level of linkage with their pandemic influenza preparedness plans. Only five of them had costed and budgeted the plan. Six countries had disseminated the plan, generally via the ministry of health website (Bangladesh, Bhutan, Indonesia, Maldives, Sri Lanka and Thailand).

All countries reported having a mechanism for releasing funds for a potential Ebola virus disease importation or outbreak (task A3), including eight whose mechanism relied on a legislative framework that was not necessarily associated with disaster situations. However, five countries expressed difficulties in releasing funds dedicated to preparedness activities, two of which struggled with major bottlenecks in funding and had asked WHO for financial support.

Only Bhutan, Maldives and Thailand had introduced a bonus system or hazard pay for health and non-health professionals in high-risk assignments, or compensation in case of infection or death (task A4). Others countries had it only for health-care professionals or had some sort of compensation based on promotion or choice of transfer. Bangladesh and Nepal reported no special plan for staff motivation or compensation for high-risk assignments.

### Risk assessment

Risk assessment is a core capacity requirement of the 2005 IHR. It provides objective information needed for decision-making and adequate risk-based preparedness and response. Our review showed that risk assessment (i.e. evaluating the likelihood of Ebola virus disease being imported or introduced into a non-affected country) had been formally or informally conducted in six countries (Bhutan, Indonesia, Maldives, Sri Lanka, Thailand and Timor-Leste). Of these, most had conducted a risk assessment only once at the early phase of preparedness, rather than as a continuous evaluation, and most relied on the results of the regular WHO global risk assessments in which country-specific recommendations are limited. Only Sri Lanka and Thailand used risk assessment for preparedness by identifying several scenarios of Ebola virus disease to be addressed in the preparedness process. At the time of our review, no reports of such risk assessment were documented and none of the countries had developed formal risk assessment procedures or a manual (task B1).

### Coordination

High level authorities of all countries were committed to Ebola virus disease preparedness planning. Coordination mechanisms and systems relied on existing structures (a committee or task force) that had been developed during the avian influenza pandemic threats (e.g. A/H5N1, A/H7N9) or the 2009 A/H1N1 influenza pandemic. All of these committees were multisectoral and multilevel and led by high-level health authorities (task C1). Usually a technical subcommittee had been set up to develop and implement the Ebola virus disease plan, backed by a multidisciplinary expert committee (task C2). The incident management structure, with roles and responsibilities defined, were detailed in the Ebola virus disease preparedness plans (task C3). Indonesia, Myanmar and Sri Lanka had encouraged committees at the subnational level to develop preparedness plans.

Countries had different understandings of the functions of an emergency operating centre, such as where a centre should be located and whether they needed several centres or one comprehensive emergency operating centre encompassing all types of response. Also, the potential to use such a centre as a centre for data management and analysis was often overlooked. While many epidemiology and surveillance departments did not have a functional emergency operating centre or a definite location for it at the time of the review, all ministries of health had such a centre handled by the ministry’s disaster management department (task C4).

### Surveillance 

Among the reviewed countries, Sri Lanka and Thailand fully satisfied the effectiveness criteria of an early warning system (task D1) and capacity to identify potential incubating travellers (i.e. travellers who had visited Ebola-affected countries) for medical follow-up (task I6).

Most countries use an Internet-based system to report diseases. Bangladesh, Myanmar, Nepal and Timor-Leste had no national system of immediate reporting (e.g. legally binding system of notifiable diseases). Others relied solely on sentinel public hospitals and tally sheets to report cases in an aggregated manner. Indonesia and Nepal reported insufficient focus on raising awareness about Ebola virus disease among clinicians from the private and public sectors (task D2).

In general, a country’s surveillance/epidemiology unit should coordinate the 21-day follow-up of at-risk travellers returning from affected countries from the list provided by airport health offices. The system was in place in all countries and appeared functional in most. Event-based surveillance was acknowledged by all countries to be efficient for detecting clusters of unknown events in the community or hospitals (task D4 and D5). 

### Laboratory

With respect to laboratory preparedness, all countries had at least one national reference laboratory. Bangladesh, Indonesia, Nepal and Thailand possessed a biosafety level 3 facility; however, only two of these (in Indonesia and Thailand) were actually functional at the time of our visit. Nevertheless, all these laboratories had, or could upgrade rapidly to, biosafety level 2+ capacity if necessary (i.e. a minimum capacity that could permit inactivation of specimens and where laboratory technicians are well trained in use of personal protective equipment; task E1). While the smaller countries (Bhutan, Maldives and Timor-Leste) did not have virologists with higher degree qualifications, all countries had laboratory technicians skilled in polymerase chain reaction (PCR) testing methods, who could process samples in biosafety level 2+ conditions; this was a direct result of the development of national influenza surveillance centres (task E3).

Only Bangladesh, Indonesia and Thailand had developed a molecular technique for Ebola virus disease diagnosis; all three had identified suspected Ebola virus disease cases in the past year. Others had stand-by arrangements with a courier company to transport specimens, and expected to rely on the WHO Regional Office for South-East Asia to assist in directing the specimens to a suitable reference laboratory (task E2).

### Investigation

All countries had integrated the concept of rapid response teams into their response to a public health event. All had such teams at the central and subnational level and were using a multisectoral and multidisciplinary approach (task F1). Some countries conducted extensive training or simulations regarding an Ebola virus disease outbreak, followed by refresher courses; the primary trainings were on personal protective equipment and information about Ebola virus disease. Some countries (Bhutan, Sri Lanka and Thailand) had developed a more cost–effective approach, which involved extensive training and simulations at the central level and only providing instructions to the subnational level. Training would be rolled out to the subnational rapid response teams should the risk of introduction or spread of Ebola virus increase (tasks F2–5). 

Smaller countries (Bhutan, Maldives and Timor-Leste) reported issues related to insufficient skills among rapid response team staff, and a high turnover of staff, which meant that refresher courses needed to be conducted more frequently.

### Infection control

All countries had designated at least one national reference hospital for management of patients with Ebola virus disease; all but one had evidence that Ebola disease information was disseminated as part of educational activities among health and non-health hospital staff. Indonesia, Sri Lanka and Thailand conducted training extensively within the hospital or in many designated hospitals using a cascade training approach or mobile training teams (task G1).

Our review found that only Indonesia, Maldives, Sri Lanka and Thailand showed evidence of operational readiness to isolate and manage a suspected or confirmed Ebola virus disease case (i.e. had suitable isolation rooms ready to accommodate and treat patients; staff trained in Ebola virus disease response; appropriate supplies; and systems for management of clinical and human waste). Of these, one country recognized that it would face difficulties if several cases were to be isolated or if contacts needed to be quarantined (task G2).

Many of the visited hospitals had primarily developed a system for separating referred suspected Ebola virus disease patients from other patients. Triage procedures for use by health-care personnel for suspected walk-in patients at an emergency department were poorly planned or adopted. Comprehensive exercises had been conducted in the visited hospitals in five countries (Bhutan, Indonesia, Maldives, Sri Lanka and Thailand; task G3).

All but two countries acknowledged having limited clinical expertise for managing an Ebola virus disease case; participants reported that most infectious disease physicians had self-trained using WHO and other international institutions’ clinical management guidelines, and few of them had been to the regional training on Ebola clinical management held in Bangkok, Thailand in March 2015. All but one country had prepared a telephone hotline support system connecting health-care providers with a team of clinicians with expert knowledge (task G4).

### Communication

Capacity for raising public awareness and social mobilization about Ebola virus disease was high across the countries. Thanks to high Internet coverage, countries could easily disseminate information and WHO guidelines about Ebola virus disease to the subnational level. Most countries acknowledged gaps in risk communication and requested support for further strengthening of this. All countries reported having functioning communication coordination mechanisms involving all government sectors and other stakeholders and these had been strengthened and tested during the avian influenza threats and the recent pandemic influenza periods.

### Points of entry

Our visits to international airports in each country found a high level of awareness about the threat posed by the possible arrival of Ebola-infected patients. WHO has recommended that airport staff should identify international travellers exhibiting signs and symptoms of Ebola virus disease, or with a history of exposure to Ebola virus, and provide a coordinated response on arrival.^6^ While some airports were not up to standard or poorly equipped (e.g. without an isolation or holding-area facility), there was close collaboration between the airport authorities and the health authorities in all countries. Mechanisms for sharing information about at-risk travellers between the surveillance department and the health offices at airports were in place and appeared to be functional (task I5). Specific emergency plans for importation of Ebola virus disease or Middle East respiratory syndrome (MERS) coronavirus were tested by undertaking drills that encompassed detection of a suspected Ebola virus disease case and transfer from the airport to the reference hospital (with whom stand-by arrangements had been made beforehand) (task I1). 

Communication to travellers is paramount so that any at-risk travellers can report to the health authorities for medical screening and a 21-day follow-up. Nevertheless, we felt that in some airports in Bhutan, Indonesia, Maldives and Nepal, the authorities had recently lowered their guard on communication and advice to travellers, probably due to Ebola preparedness fatigue. This situation may increase the risk of a traveller with incubating Ebola virus entering the country and not reporting voluntarily (particularly those travelling from a non-affected third country; task I6).

## Discussion

All of the countries that we reviewed have committed to Ebola virus disease preparedness and response planning. Preparedness was most advanced on the following key components: multilevel and multisectoral collaboration and coordination structures; multidisciplinary rapid response teams at the central level; capacity for public communication and social mobilization; some level of preparedness in international airports; training on personal protective equipment; and laboratories with molecular diagnostic capacity. Planning was triggered in all countries after WHO declared Ebola virus disease as a public health emergency of international concern in 2014. The Ebola preparedness plans tended to rely on generic structures previously established for influenza pandemics in the countries.^7^ Effectiveness in implementing Ebola virus disease preparedness can therefore be interpreted as a return on investment in IHR capacities.^8^ This underscores the fundamental importance of the IHR mechanism for global health security. 

Our study provides not only an indication of Ebola disease preparedness but also a measure of countries’ progress towards meeting IHR core capacity requirements. By the end of 2015 only Thailand and Indonesia have reported to WHO that they have met IHR requirements in 2014. Several improvements are needed if all countries in the WHO South-East Asia Region are to comply with the IHR.

First, efforts are needed to strengthen risk assessment capacity across the region. Risk assessment, when conducted, was limited in scope in most countries, because processes, risk questions and recommendations were unclear or not made available. There was a limited use of risk assessment, with its potential to evaluate system vulnerabilities in a transparent way and to identify process and knowledge gaps.^9^^,^^10^

Second, the risk communication capacity of countries was also weak: unsurprisingly, as this is closely linked with risk assessment.^11^^–^^13^ Most countries had deficiencies in this area, and recognized difficulties in developing their risk communication strategic and action plan.

Third, preparedness efforts to ensure continuity of care for potential Ebola cases, which include danger pay for staff, were not optimal in many of the countries. Most participants in the discussions felt that keeping health-care staff on the job if an Ebola case were suspected would be a challenge. Only Indonesia and Thailand had experience in handling a highly contagious disease (e.g. H5N1 influenza virus infection since 2004).

Fourth, while all the countries possessed indicator-based and event-based surveillance, as required by the IHR,^14^^,^^15^ most acknowledged that a timely and sensitive early warning system was difficult to achieve. This was due to several factors: slow collection of data from a limited number of sites; no case-based, immediate reporting mechanism; and limited capacity to process and analyse data. Further investments in automated surveillance that rapidly collects and analyses large amounts of data may be needed.^16^

Fifth, most countries had not attempted to introduce molecular techniques for Ebola virus disease diagnosis, even though they had PCR testing capacity for other viruses (e.g. MERS-coronavirus or influenza viruses) and had laboratory capacity at the minimum biosecurity level for Ebola virus inactivation (biosafety level 2+ or 3).^17^ In some of these countries, experience with a handful of suspected patients (later found to be negative) showed that patients’ PCR results took 5–7 days to be returned from reference laboratories in other countries. This delay highlights a need for in-country capacity for Ebola virus disease diagnosis, supported by stand-by arrangements with global WHO collaborating centres.^17^

Other challenges that needed improvement in the countries included several elements that were prominent in the 2013–2016 West African Ebola virus disease epidemic:^3^ advice to inbound travellers; adequate isolation rooms; appropriate infection control practices; emergency department triage systems in general hospitals; contact tracing; and danger pay to health-care workers to ensure continuity of care. Staff fears about Ebola virus contagion are important to address, as even the best plans can fail if there is absenteeism and disruptions in supporting services and supplies.

Finally, in some countries, particularly the smaller ones, substantial shortfalls in preparedness were revealed concerning: accommodating a surge of cases in health-care facilities; testing for multiple cases and contacts; and mobilizing staff for contact tracing. Countries need to be prepared for a scenario that rapidly overwhelms the capacity of health authorities. They should therefore consider detailed surge capacity planning that includes stand-by arrangements with other ministries (e.g. defence or interior) and civil society or international partners.

Our findings have some limitations. First, the results are just a snapshot of each country’s situation: a status that is dynamic and can improve or deteriorate. Second, findings were based on a broad review of procedures rather than a quality analysis of the documents or direct observations of performance. An overall high level of readiness should be interpreted as indicating that the country is taking steps to ensure that its plan is truly operational and that the planned activities are actionable. Third, our assessment indicators were adapted from the WHO Ebola preparedness checklist,^3^ but, due to time constraints, have not been formally piloted. The choice of indicators and the scoring system can be debated. For example, due to time constraints we chose not to study preparedness on specific logistics of Ebola virus disease from the WHO checklist.^3^ Instead we focused on the main pillars of the IHR. Rather than evaluating and comparing countries, our joint WHO and health ministry approach aimed to help countries to prioritize and formally document their most urgent needs to enhance preparedness and response within their health security system. We appreciate that Sri Lanka has made the report publicly available,^18^ which is one of the goals of the review. We hope that other countries are encouraged to use a similar transparent and constructive process whereby WHO and national participants work together in interactive sessions to reach a consensus with clear justifications. Transparency and consensus were adopted by WHO’s joint external evaluation in 2016 to monitor IHR compliance and help attract and direct resources to where they are needed most.^19^

This study has provided a general picture of comparative strengths and weaknesses across various aspects of Ebola disease preparedness that are also key components of the IHR core capacity requirements. Further strengthening of IHR capacities must involve testing the functionality of preparedness and response systems. An IHR monitoring and evaluation mechanism is needed that incorporates joint assessment processes, repeated simulation exercises and risk assessment processes that look into system vulnerabilities. Many countries have a limited ability to address every type of hazard or large-scale event. IHR-related planning should therefore include detailed stand-by arrangements between countries and with WHO on areas of vulnerability.
